# Monitoring determinants of the prevalence of child malnutrition in Brazil according to indicators of the 2030 Agenda in the year 2022

**DOI:** 10.1590/1980-549720250001

**Published:** 2025-01-31

**Authors:** Eliete Costa Oliveira, Ana Karina Teixeira da Cunha França, Sueli Ismael Oliveira da Conceição, Victor Nogueira da Cruz Silveira, Maylla Luanna Barbosa Martins Bragança, Alcione Miranda dos Santos

**Affiliations:** I Universidade Federal do Maranhão, Graduate Program in Public Health, Biological and Health Sciences Center - São Luis (MA), Brazil.; II Universidade Federal do Maranhão, Biological and Health Sciences Center, Department of Physiological Sciences - São Luis (MA), Brazil.

**Keywords:** Child nutrition disorders, Sustainable development, Social determinants of health, Maternal and child health

## Abstract

**Objective:**

To select indicators of the Sustainable Development Goals (SDGs) that determine child malnutrition (CM) in Brazil and to monitor the achievement of SDG targets by region in 2022.

**Methods:**

This is a cross-sectional, ecological study that used the Brazilian Sustainable Development indices and analyzed the 100 SDG monitoring indicators in the 5,570 Brazilian municipalities. A decision tree was created and sensitivity analysis was performed to predict CM determinants. Data were analyzed using the χ^2^ test at 5% significance level. Descriptive analyses and the decision tree were carried out using the R software.

**Results:**

The CM determinants according to percentage, most affected regions of the country, and impact order were: illiteracy in the population aged ≥15 years (Northeast), insufficient prenatal care (North), low birth weight (South), young women aged 15-24 years who neither study nor work (North and Northeast), and employed population aged 10-17 years (South). We observed an individual and cumulative effect on the CM prevalence, ranging from 1.73 to 15.1%, in Brazilian municipalities according to the occurrence and overlap of these indicators.

**Conclusion:**

The results denote that Brazil will not achieve the intended reduction of CM by 2025. There must be substantial investments in education and health mainly aimed at the maternal and child population and especially in the North and Northeast regions.

## INTRODUCTION

Child malnutrition (CM) is characterized by a deficiency of basic nutrients that culminates in weight and height deficits^1^, and is considered a public health issue in middle- and low-income countries^2^. Indices — such as weight for age (W/A) and height for age (H/A) — were adopted by the United Nations (UN) for the diagnosis of CM, with W/A being a measure of recent nutritional status and H/A, a history of nutrition and health conditions since birth, making them important requirements for assessing the country's socioeconomic and political situation^3-5^.

In 2000, the Millennium Development Goals (MDGs) were established by UN member states, and one of the targets was to reduce the global prevalence of stunting in children aged <5 years by half, by the year 2015^3^. Since then, food and nutritional security has become the target of public policies to combat poverty and CM in Brazil^6^, and the prevalence of CM has reduced from 13.5% in 1996 to 6.8% in 2007 — attributed to the expansion of health coverage, education, sanitation, and increased family income^7^.

Following the success in achieving many MDGs targets, in 2012, the 2030 Agenda was established, with 17 Sustainable Development Goals (SDGs), in force as of 2016^8,9^. One of the international targets is to end all forms of malnutrition by 2030, and to achieve the international targets on stunting (reduce it by 40%) and underweight (reduce it and maintain <5%) in children aged <5 years by 2025^9^. National targets were adjusted by Brazilian experts to H/A <3%^10,11^. It should be noted that low W/A is an acute condition that can change frequently and rapidly, which makes it difficult to generate reliable trends over time^12^, while stunting refers to a chronic condition, regardless of the child's ethnicity, socioeconomic status, and type of feeding^13^.

The socioeconomic and political crisis that took place in the country in 2014 impacted the increase in poverty, the adoption of tax austerity measures, the increase in food prices, and the reduction of social protection measures for the most socioeconomically vulnerable groups, contributing to the increase in CM^14^. In 2019, the prevalence of stunting reached 13.4%, reducing to 11.7% in 2022, with the lowest prevalence values observed in the South (9%) and Southeast (11.5%), and the highest in the North (15.4%) and Northeast (12.9%), according to data from the Food and Nutrition Surveillance System (*Sistema de Vigilância Alimentar e Nutricional* - SISVAN)^15^. This system presents data on the population served by Primary Health Care (PHC) and beneficiaries of income transfer programs^5^, as nutritional monitoring is part of their conditionalities^16^.

There is a need to monitor SDG indicators to achieve the target of reducing CM by 2025, as it is a persistent problem in Brazil. Furthermore, CM presents regional disparities and consists of a complex process influenced by intrinsic (physiological) and extrinsic (food, housing, environment, family income, parents' education and access to goods, essential services^17^, and maternal and child care^18^) causes that integrate several SDG indicators^19^. In Brazil, there are no studies whose authors have investigated the interactions between SDG indicators and the prevalence of CM.

The application of robust statistical methods, such as decision and regression trees (DRT)^20^, enables to identify the interaction of different factors with the prevalence of CM. From this analysis, we can identify the SDG indicators that determine CM in children aged <5 years in Brazilian municipalities as well as its individual and cumulative effects on this prevalence.

In this study we aimed to select the SDG indicators that determine CM in Brazil using the DRT in 2022 and monitor the achievement of CM targets, by region. The findings of this study may provide information that guides decision-making in the implementation of social and health public policies.

## METHODS

This is an ecological study with the use of data from the Sustainable Development Index of Cities — Brazil (*Índice de Desenvolvimento Sustentável das Cidades -* IDSC-BR) to monitor the implementation of the SDGs. IDSC-BR uses data produced by national sources, such as the Brazilian Institute of Geography and Statistics (IBGE), the Department of Informatics of the Brazilian Unified Health System (Datasus), the Anísio Teixeira National Institute of Studies and Educational Research (*Instituto Nacional de Estudos e Pesquisas Educacionais Anísio Teixeira* - INEP), the Institute for Applied Economic Research (*Instituto de Pesquisa e Economia Aplicada* - Ipea); information-producing agencies; and executors of government policies linked to the UN^11^. The data used in this study were published in 2022 and are available on the IDSC-BR website (https://www.cidadessustentaveis.org.br/).

In this study, the 5,570 Brazilian municipalities^21^ were evaluated by the 100 indicators of the 17 SDGs, including CM, whose data come from SISVAN^5^. The dependent variable was the prevalence of CM in children aged <5 years, measured by the H/A index, while the independent variables were the other 99 SDG indicators (Chart 1).

**Chart 1:** Indicators of the Sustainable Development Goals and respective cut-off points.

Sustainable Development Goal	Indicator	Target value (%)	Green threshold (%)	Red threshold (%)	Lower threshold (%)
1	Families registered in the Cadastro Único [Single Registry] (a Federal Government initiative that gathers information on Brazilian families in situation of poverty and extreme poverty) for social programs (%)	96	87	64	48
1	Percentage of people registered in the Cadastro Único who receive Bolsa Família	96.6	80.5	42.82	22.96
1	Percentage of people below the poverty line in the Cadastro Único after Bolsa Família	0	21.48	41.59	92.8
1	People with income of up to 1/4 of the minimum wage (%)	0.18	5.74	4.45	15.45
2	Childhood obesity (%)	0	5	10	20
2	Low birth weight (%)	0	6	11	13
2	Child malnutrition (%)	0	1	3	5
2	Family farming producers with support from PRONAF (%)	100	75	55	6
3	Vaccine coverage (%)	100	95	60	40
3	Infant mortality (children under 1 year of age) (one thousand live births)	0	12	19	45
3	Maternal mortality (one thousand live births)	0	0.61	3.21	6.7
3	Child mortality (children under 5 years of age) (one thousand live births)	0	25	37	50
3	Neonatal mortality (children aged 0 to 27 days) (one thousand live births)	0	12	20	36
3	Municipal health budget (in BRL, per capita)	4,680	1,300	476	395
3	Population served by family health teams (%)	100	86	60	0
3	Insufficient prenatal care (%)	0	10	38	59
3	Health Centers (one thousand inhabitants)	1.89	0.55	0.15	0
3	Life expectancy at birth (years)	79	75	71	67
3	Pregnancy in adolescence (%)	0	9.98	23.46	30.81
4	Basic Education Development Index (*Índice de Desenvolvimento da Educação Básica* - IDEB) - initial years (IN)	8.98	6.65	4.67	3.8
4	Young people with complete high school by the age of 19 years (%)	100	70	42	5
4	Illiteracy in the population aged 15 years and over (%)	0	3	17	30
4	Children and young people aged 4 to 17 years at school (%)	100	95	87	82
5	Young women aged 15 to 24 years who neither study nor work (%)	0.83	20.46	39.4	47.06
5	Gender pay gap (women's salary/men's salary)	1	0.9	0.6	0.5
6	Diseases related to inadequate environmental sanitation (100 thousand inhabitants)	0	136.21	367.4288361	967.12
6	Population served by water service (%)	100	85	53	0
6	Population served by sanitary sewer (%)	100	70	50	0
6	Population served with household collection of municipal solid waste (%)	100	80	60	0
7	Households with access to electricity (%)	100	99	90	80
8	Employed population aged 10-17 years (%)	0	7.59	25.93	41.32
8	GDP per capita (BRL per capita)	56,000	38,000	23,000	7,300
8	Unemployment (rate)	0	3	10.27	15.57
9	Public investment in infrastructure as a proportion of GDP (%)	15	10	5	0.6
9	Share of jobs in knowledge- and technology-intensive activities (%)	43.28	14.3	1.92	0
10	Municipal income appropriated by the 20% poorest (%)	20	10	7	1.5
10	Gini coefficient	0.275	0.3	0.4	0.63
10	Infant mortality ratio (Black/non-Black)	1	1	1.44	2.08
10	Access to primary health care equipment	0	2	30	100
11	Population living in substandard settlements (%)	0	0.8	5	22
11	Households in slums (%)	0	1.04	5.55	13.12
12	Household waste per capita (Ton/Inhabitant/Year)	1	1.5	2	3.2
17	Public investment (BRL per capita)	2,253.88	563.255	239.105	60.79
17	Total revenue collected (%)	51.35	19.73	3.9	1.19

PRONAF: National Program for Strengthening Family Agriculture (*Programa Nacional de Fortalecimento da Agricultura Familiar*); GDP: gross domestic product.

According to IDSC-BR, the recommended nomenclature and categorization for SDG indicators are: target value (represented by the color green), green threshold (in yellow), red threshold (in orange), and lower threshold (in red). It also considers achieved targets to be the combination of the target value and green threshold, which were, didactically, represented in this study only by the color green; and unachieved targets to be the combination of the red threshold and the lower threshold, represented by the color red^11^. The values established by the SDGs to assess the prevalence of CM were: <1% (target value); 1 to <3% (green threshold); 3 to <5% (red threshold); and ≥5% (lower threshold)^11^ (https://www.cidadessustentaveis.org.br/methodology).

In the descriptive analysis, categorical variables were presented in absolute and relative frequencies, while continuous variables were presented as means and standard errors of the sample mean (SE).

To select the indicators determining the prevalence of CM, the DRT was used^20^. Once the tree was created, the sensitivity to predict the main independent variables determining CM was calculated. DRT is a method that divides data into segments that are as homogeneous as possible in relation to the outcome variable (prevalence of CM). A node is considered homogeneous when all cases have the same value for the outcome based on a specific determinant^20^. The χ² test was performed to verify the statistical significance of these correlations and a 5% significance level was adopted. Descriptive analyses and the decision tree were performed using the R software, version 4.3.0.

This study does not require an Informed Consent Form or submission to the Research Ethics Committee as it uses data from IDSC-BR, which do not include confidential information and are freely accessible. Ethical issues, guidelines, and standards that regulate research in Brazil were respected.

## RESULTS

**Figure 1:**
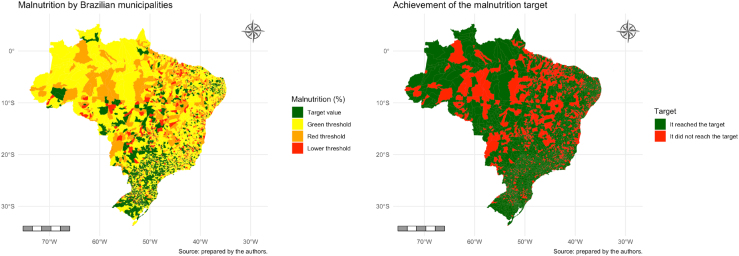
Distribution of child malnutrition and achievement of targets established by the Sustainable Development Goals in Brazilian municipalities, according to the classification of the Brazilian Sustainable Development Indices, 2022.

The distribution of CM in Brazil, according to the SDG color panel, and the achievement of its targets are presented in Figure 1. The concentration of municipalities that reached the target for the prevalence of CM in the South and Southeast regions is clear: 76.5% of municipalities, with 16.2% classified as red threshold and 7.3% as lower threshold (Table 1).

**Table 1:** Characterization of child malnutrition and its determinants, in Brazil and regions, according to the Brazilian Sustainable Development Indices, 2022.

Indicators		Thresholds SDGs	Brazil n=5,570*	Midwest n=467*	Northeast n=1,794*	North n=450*	Southeast n=1,668*	South n=1,191*
Child malnutrition (%)								
	Target value	≥0 and <1	1,776 (31.9)	129 (27.6)	309 (17.2)	61 (13.6)	600 (36)	677 (56.9)
	Green threshold	≥1 and <3	2,484 (44.6)	179 (38.4)	947 (52.8)	241 (53.6)	764 (45.8)	353 (29.6)
	Red threshold	≥3 and <5	903 (16.2)	94 (20.1)	393 (21.9)	111 (24.6)	211 (12.6)	94 (7.9)
	Lower threshold	≥5	407 (7.3)	65 (13.9)	145 (8.1)	37 (8.2)	93 (5.6)	67 (5.6)
Illiterate population aged ≥15 years								
	Target value	≥0 and <3	102 (1.8)	0 (0)	0 (0)	0 (0)	16 (1)	86 (7.2)
	Green threshold	≥3 and <17	3,256 (58.5)	411 (88.2)	125 (7)	237 (52.8)	1,406 (84.3)	1,077 (91)
	Red threshold	≥17 and <30	1,532 (27.5)	55 (11.8)	1,032 (57.5)	188 (41.9)	232 (13.9)	25 (2.1)
	Lower threshold	≥30	675 (12.2)	0 (0)	637 (35.5)	24 (5.3)	14 (0.8)	0 (0)
Insufficient prenatal care (%)								
	Target value	≥0 and <10	614 (11)	38 (8.1)	85 (4.7)	4 (0.9)	216 (12.9)	271 (22.7)
	Green threshold	≥10 and <38	4,164 (74.8)	362 (77.6)	1,337 (74.6)	195 (43.3)	1,384 (83)	886 (74.4)
	Red threshold	≥38 and <59	653 (11.7)	65 (13.9)	312 (17.4)	178 (39.6)	66 (4)	32 (2.7)
	Lower threshold	≥59	139 (2.5)	2 (0.4)	60 (3.3)	73 (16.2)	2 (0.1)	2 (0.2)
Low birth weight (%)								
	Target value	≥0 and <6	1,243 (22.3)	144 (30.8)	438 (24.4)	129 (28.7)	275 (16.5)	257 (21.6)
	Green threshold	≥6 and <11	3,476 (62.4)	268 (57.4)	1,191 (66.4)	290 (64.4)	1,057 (63.4)	670 (56.2)
	Red threshold	≥11 and <13	439 (7.9)	29 (6.2)	102 (5.7)	14 (3.1)	187 (11.2)	107 (9)
	Lower threshold	≥13	412 (7.4)	26 (5.6)	63 (3.5)	17 (3.8)	149 (8.9)	157 (13.2)
Young women aged 15-24 years who neither study nor work (%)								
	Target value	≥0.83 and <20.46	850 (15.4)	19 (4.1)	18 (1)	4 (0.9)	189 (11.3)	620 (52.2)
	Green threshold	≥20.46 and <39.4	3,876 (69.6)	377 (80.9)	1,262 (70.3)	323 (71.9)	1,374 (82.4)	540 (45.5)
	Red threshold	≥39.4 and <47.06	699 (12.5)	59 (12.6)	423 (23.6)	97 (21.6)	97 (5.8)	23 (1.9)
	Lower threshold	≥47.06	140 (2.5)	11 (2.4)	91 (5.1)	25 (5.6)	8 (0.5)	5 (0.4)
Employed population aged 10-17 years								
	Target value	≥0 and <7.59	547 (9.8)	19 (4.1)	294 (16.4)	37 (8.2)	178 (10.7)	19 (1.6)
	Green threshold	≥7.59 and <25.93	4,317 (77.5)	413 (88.4)	1,397 (77.9)	376 (83.6)	1,424 (85.4)	707 (59.4)
	Red threshold	≥25.93 and <41.32	566 (10.2)	34 (7.3)	97 (5.4)	34 (7.5)	62 (3.7)	339 (28.4)
	Lower threshold	≥41.32	140 (2.5)	1 (0.2)	6 (0.3)	3 (0.67)	4 (0.2)	126 (10.6)

* number of municipalities. SDGs: Sustainable Development Goals; Target value: value that reflects the best performance of the municipality, established as the SDG target for the referred indicator; Green threshold: value of the indicator, from which it is considered that the municipality has reached the SDG target; Red threshold: value that denotes distance from achieving the target intended by the municipality for the SDG indicator; Lower threshold: value that reflects the worst performance of the municipality for the indicator under study.

The South region had 86.5% of municipalities that reached the CM reduction target, followed by the Southeast region, with 81.8%. In the Northeast, North, and Midwest regions, the percentages of municipalities that reached the target were 70, 67.1, and 66%, respectively (Table 1).

**Figure 2:**
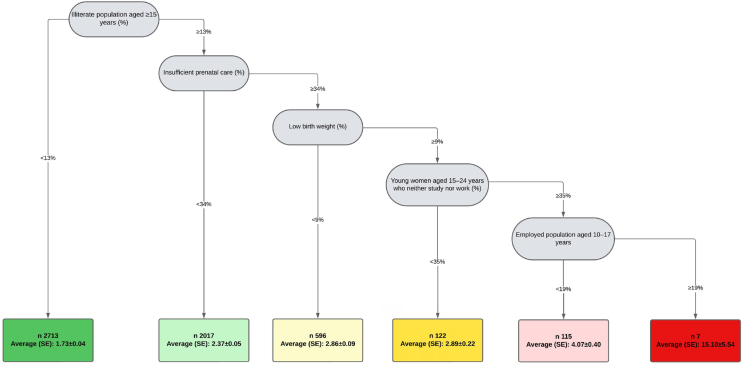
Decision and regression tree and the correlation between the indicators of the Sustainable Development Goals determining child malnutrition. Brazil, 2022.

The following indicators were selected by the DRT as determinants of CM in Brazil: illiteracy in the population aged ≥15 years (Illiterate population aged ≥15 years), insufficient prenatal care (IPC), low birth weight (LBW), young women aged 15 to 24 years who neither study nor work (Women aged 15-24 years neither-nor), and employed population aged 10 to 17 years (Employed population aged 10-17 years). We estimated the cumulative effect of the increase in these indicators on the prevalence of CM (Figure 2).

The prevalence of municipalities that achieved the target of reducing illiteracy among people aged ≥15 years was 60.3% and the target of exceeding the IPC was 85.8%. The South, Midwest, and Southeast regions had the highest number of municipalities that reached the target. Nonetheless, 93% of municipalities in the Northeast did not reach the target of reducing illiteracy, and 55.8% of municipalities in the North did not reach the target of exceeding the IPC (Table 1).

In Brazil, 84.7% of municipalities reached the target of exceeding LBW. The South and Southeast regions had the highest number of municipalities that did not reach the target: 22.3 and 20.1%, respectively (Table 1).

Among Brazilian municipalities, 85% achieved the target of reducing the indicator Women aged 15-24 years neither-nor. The Northeast and North regions had the highest number of municipalities that did not reach the target for this indicator (28.7 and 27.2%, respectively). 87.3% of municipalities reached the target of reducing the Employed Population aged 10-17 years, and the South region had the highest percentage of municipalities that did not reach the target (39%) (Table 1).

According to the DRT analysis, in 2,713 municipalities, in which the percentage of Illiterate population aged ≥15 years was <13%, the prevalence of CM was 1.73% (SE±0.04). In 2,017 municipalities, where the percentage of Illiterate population aged ≥15 years was ≥13%, associated with IPC <34%, the prevalence of CM rose to 2.37% (SE±0.05). In 596 municipalities, where the percentages of Illiterate population aged ≥15 years were >13%, IPC ≥34%, and LBW <9%, the prevalence of CM was 2.86% (SE±0.09). In 122 municipalities that, in addition to Illiterate population aged ≥15 years >13%, IPC ≥34%, LBW ≥9%, Women aged 15-24 years neither-nor accounted for <35%, the prevalence of CM increased to 2.89% (SE±0.22) (Figure 2).

In 115 municipalities that presented >13% of Illiterate population aged ≥15 years, IPC ≥34%, LBW ≥9%, Women aged 15-24 years neither-nor ≥35%, and Employed population aged 10-17 years <19%, the prevalence of CM was 4.07% (SE±0.40). In seven municipalities, where Employed population aged 10-17 years was ≥19% and all other inequities remained, the prevalence of CM increased to 15.4% (SE±5.54) (Figure 2).

## DISCUSSION

In this study we showed that the SDG target for reducing CM was achieved by 76.5% of Brazilian municipalities, with the South and Southeast regions standing out. The Midwest had the highest percentage of municipalities that did not reach this target. Five SDG indicators were selected as determinants of the CM according to percentages and order of impact (according to the emergence of the indicator in the DRT): Illiterate population aged ≥15 years, IPC, LBW, Women aged 15-24 years neither-nor, and Employed population aged 10-17 years.

The fact that the Midwest region has a higher prevalence than the North raises questions about underreporting and incomplete data in this region, which are common in national surveys and can mask results and impact decision-making^22^.

The prevalence of CM due to H/A in Brazil, according to data from the National Demographic and Health Survey (*Pesquisa Nacional de Demografia e Saúde* - PNDS/2007), was 6.8%^7^, reaching the target established by the MDGs^3^. However, data from SISVAN showed higher values, ranging from 15.1% (2008) to 13.4% (2019), with a small reduction in 2022 (11.7%), being greater in the North (15.4%) and Northeast (12.9%)^15^. Conversely, authors of the Brazilian National Survey on Child Nutrition (*Estudo Nacional de Alimentação e Nutrição Infantil* - ENANI/2019), representative of the Brazilian population, demonstrated a prevalence of 7% of low H/A, being higher in the North (8.4%), Southeast (7.3%), and South (7%)^23^.

Due to the gap between PNDS/2007 and ENANI/2019, since 2008, SISVAN data have been used to monitor these targets^5^. SISVAN presents data from all regions and enables the study of the population served by PHC^24^ and beneficiaries of the Bolsa Família Program (a cash transfer program of the Brazilian government)^5,16^. Subsequently, data from ENANI/2019 began to be incorporated into SDG monitoring reports to analyze these indicators^25^.

Regardless of regional differences, the socioeconomic and political crisis that has taken hold in Brazil since 2014 has had an impact on the increase in poverty; increased food prices; the adoption of tax austerity measures; and the reduction of social protection measures, contributing to the increase in CM^14^.

Despite the COVID-19 pandemic in 2020, the protective measures adopted by the government, at all levels — through the creation of Emergency Aid; Emergency Employment and Income Maintenance Program^26^; and specific financial incentives for PHC to combat malnutrition, focused on children and pregnant women —, contributed to mitigating the effect of food insecurity and attenuating the impact of the pandemic on CM^27^.

The severity of functional illiteracy was evident in the Northeast and North, where a large proportion of municipalities did not reach the target, in contrast to other regions. Authors of a study conducted in southern Africa found that the prevalence of CM varied between 3.4 and 30.2%, and was associated with illiteracy^28^. The latter can trigger fewer job opportunities, lower income, and difficulty in acquiring food and accessing health services, contributing to CM^7^.

The target of reducing illiteracy was not achieved by the National Education Plan 2014-2024 due to the non-implementation of public education policies aimed at the public aged ≥15 years^29^. Furthermore, no mechanisms were established to make working hours compatible, nor was there a specific income transfer program^30^. Only in 2024 the Pé de Meia [Nest Egg] Program (a financial-educational government support in the form of savings aimed at promoting student retention and school completion) was instituted, aimed at students aged 14 to 24 years in public schools and those belonging to the Youth and Adult Education (EJA) category, aged 19 to 24 years^31^.

The target of exceeding the IPC was achieved by many municipalities in Brazilian regions, with the exception of the North region. Prenatal coverage in Brazil accounted for 89% from 2013^32^ to 2019^33^. It is worth noting that the North has a higher level of precarious services, low prenatal coverage, and worse quality indicators^34^, related to territorial coverage, difficulty in accessing health services, and low retention of professionals in the region^35^.

Nutritional monitoring during prenatal care is essential to prevent inadequate weight gain and control complications^36^ that may predispose the pregnant woman to premature birth and the child to intrauterine growth restriction (IUGR) and LBW^37^. Pregnant women should be advised on breastfeeding and adequate nutrition to prevent CM^38^.

In this research, a high frequency of Brazilian municipalities in the North, Northeast, and Midwest exceeded the LBW target; this did not occur in the South and Southeast. The prevalence of LBW is 37.7% in the South and 13.5% in the Southeast^39^.

The LBW paradox was observed in the North and South regions. This can be attributed to the improvement in prenatal care in more developed regions with a concomitant reduction in the prevalence of stillbirths and an increase in LBW^40^, as observed in the South region. We observed the opposite in the North, with a lower prevalence of LBW and a high percentage of municipalities with IPC. Possible explanations would be the high rates of underreporting of live births in the North^41^ and the high rate of cesarean sections in the South^42^, related to the interruption of high-risk pregnancies, with a consequent reduction in gestational age, increase in prematurity, and viability of newborns with extremely low birth weight^43^.

Brazil has one of the highest rates of cesarean sections in the world. In 2019, 56.3% of births were cesarean sections and carried out mainly in the Midwest, Southeast, and South regions^44^, highlighting the inequalities in the country. Subsequently, researchers indicated an increasing trend in the North and Northeast, and a decline in the South and Southeast^42^. Despite the decline, the South region still has higher prevalence rates, and Brazil continues to have cesarean section rates well above the recommended level^43^.

Public policies developed since the 1980s, which culminated in the implementation of the Rede Cegonha [Stork Network] Program (a strategy of the Brazilian Ministry of Health intended at improving the care provided to women and children)^45^, were essential for improving important practices related to childbirth and the postpartum period in Brazil. Other policies and strategies related to the protection of breastfeeding and the introduction of adequate and healthy complementary feeding were paramount for protecting children's health, especially that of infants, as it is observed that complications in the neonatal period are more related to pregnancy and childbirth and, after this period, they are more related to the child's socioeconomic context^46^.

The target of reducing the Women aged 15-24 years neither-nor indicator was achieved by 85% of municipalities, with the worst results in the North and Northeast. In 2018, 23% of young people found themselves in this situation, the majority of whom were low-income women^10^. The reasons are related to cognitive abilities, domestic obligations, and the lack of public policies that can mitigate gender inequality and increase the prospects of overcoming poverty^47^. Low levels of education, low income, and teenage pregnancy are more related to IUGR and a higher risk of producing malnourished children^48,49^. Investing in the human capital^2^ of these women can contribute to better family planning and the reduction of CM^50^.

Women's education is a strong predictor of their children's health and survival, as it influences access to health services, care in situations of illness, better job opportunities, and income generation for childcare^51^. Mothers tend to manage family expenses better, directing them towards food, clothing, and school supplies^52^.

The South region is the most affected by child labor, and has a high prevalence of the Employed population aged 10-17 years indicator. Child labor can cause harm to the physical and mental health of children and adolescents, in addition to the risk of adopting unhealthy lifestyle habits and developing chronic noncommunicable diseases^53^. It also perpetuates low levels of education, income^54^, and CM^46^. Paradoxically, in the South region, this scenario may be related to the culture that considers work as an educational tool^55^, as it did not negatively impact illiteracy rates, prenatal care, gender inequalities, and CM.

In other regions, in order to increase family income, child labor contributes to high rates of student retention, withdrawal, and school dropout, requiring the implementation of policies to discourage it. Households managed only by women increase the risk of child labor when compared to those managed by men or both sexes. The higher the parents' level of education and income, the lower the risk of dropping out of school^55,56^.

The cut-off points for each CM indicator, identified by the DRT, were within the target proposed by the SDGs. Nevertheless, these contributed to the increased prevalence of CM. Furthermore, the cumulative effect (combined effect of these indicators in the order they appear in the DRT) was even more impactful in increasing the prevalence of CM.

Some researchers have also observed the cumulative effect of indicators of social and health inequities associated with CM. A higher level of maternal education is associated with a reduction in CM^57,58^ and greater adherence to prenatal care^59^. In the state of Rio Grande do Sul, an association was found between adequate prenatal care and reduced LBW^60^. In the state of Pernambuco, an association between CM and lower maternal education and LBW was observed^61^. Authors of a review carried out in the North region associated CM with low maternal education and having an illiterate father/stepfather^62^.

The complexity of the outcome of this study is contemplated through the impact that each indicator, identified as a determinant of CM, has on its prevalence, as well as through the interrelations and interdependencies that these indicators exert on the succession of events that favored the increase in the prevalence of CM in Brazil.

The regional inequalities observed were closely related to the achievement or distance from the targets of the studied indicators, which made them vulnerable to an increase in CM.

As study limitations we mention the use of the IDSC-BR database, which: considered information from years near 2022 for the indicators, as some did not provide updated information; and uses data from SISVAN to monitor CM. However, the information obtained through IDSC-BR constitutes a robust database of the Brazilian population, created from the combination of information from various entities and agencies linked to the UN^11^. Moreover, although SISVAN presents flaws in data collection, its information is considered official for statistical purposes due to its coverage and the amount of information per state^14^.

As positive aspects, it is worth highlighting that all SDG monitoring indicators, in all Brazilian municipalities — available in the database — were used in this study, enabling a broad investigation of the issue in question. In addition, DRT was used to select the indicators determining CM and establish the cutoff points in an isolated and cumulative manner.

We conclude that the SDG monitoring indicators that determine CM in Brazil, individually and together, were Illiterate population aged ≥15 years, IPC, LBW, Women aged 15-24 years neither-nor, and Employed population aged 10-17 years. The impact of these indicators on the prevalence of CM showed an average of 1.73%, when the prevalence of Illiterate population aged ≥15 years was less than 13%; up to an average of 15.1%, when all other indicators exceeded the percentages identified by the DRT. Furthermore, most Brazilian municipalities achieved the targets for the indicators selected by the DRT.

All indicators determining CM are related to poverty and social inequalities, making it difficult to achieve the target for CM in the country. It is necessary to implement multidimensional socioeconomic policies by managers at all levels, which improve, in the short and long term, the income of vulnerable families and access to education and health services, especially for adolescents/young people and women, in such a way to minimize and stop the increase in existing inequalities.

In addition, the North and Northeast regions of the country should also be prioritized in terms of investments in maternal and child health care, in which women are monitored from prenatal care, through childbirth, the postpartum period, and the first years of the child's life — consequently minimizing health risks.
