# Supplementary material for: Monitoring determinants of the prevalence of child malnutrition in Brazil according to indicators of the 2030 Agenda in the year 2022
Source: Rev Bras Epidemiol. 2025 Jan 31;28:e250001. doi: 10.1590/1980-549720250001 (PMC11809085; doi:10.1590/1980-549720250001)
Supplement: Translation

# Translation: Monitoramento dos determinantes da prevalência da desnutrição infantil no Brasil segundo indicadores da Agenda 2030 no ano de 2022

**DOI:** 10.1590/1980-549720250001.2

**Authors:** Eliete Costa Oliveira, Ana Karina Teixeira da Cunha França, Sueli Ismael Oliveira da Conceição, Victor Nogueira da Cruz Silveira, Maylla Luanna Barbosa Martins Bragança, Alcione Miranda dos Santos

**Affiliations:** I Universidade Federal do Maranhão, Programa de Pós-Graduação em Saúde Coletiva, Centro de Ciências Biológicas e da Saúde - São Luis (MA), Brasil.; II Universidade Federal do Maranhão, Centro de Ciências Biológicas e da Saúde, Departamento de Ciências Fisiológicas - São Luis (MA), Brasil.

**Keywords:** Desnutrição infantil, Objetivos do desenvolvimento sustentável, Determinantes sociais, Saúde materno-infantil

## Abstract

**Objetivo:**

Selecionar os indicadores dos Objetivos do Desenvolvimento Sustentável (ODS) determinantes da desnutrição infantil (DI) no Brasil e monitorar o alcance das metas da DI, por região, no ano de 2022.

**Métodos:**

Estudo ecológico que utilizou os índices do Desenvolvimento Sustentável no Brasil e analisou os 100 indicadores de monitoramento dos ODS nos 5.570 municípios brasileiros. Foi construída uma árvore de classificação e regressão e calculada a sensibilidade para predizer os determinantes da DI. O teste qui-quadrado foi utilizado, e adotado nível de significância de 5%. As análises descritivas e a árvore foram realizadas com o auxílio do *software* R.

**Resultados:**

Os determinantes da DI segundo percentuais, regiões do país mais afetadas e ordem de impacto foram: analfabetismo na população com ≥15 anos (Nordeste), pré-natal insuficiente (Norte), baixo peso ao nascer (Sul), mulheres jovens de 15 a 24 anos de idade que não estudam nem trabalham (Norte e Nordeste) e população ocupada entre 10 e 17 anos (Sul). Observou-se efeito individual e cumulativo na prevalência de DI nos municípios brasileiros, variando de 1,73 a 15,1%, de acordo com a ocorrência e sobreposição desses indicadores.

**Conclusão:**

Os dados indicam que o Brasil não atingirá a meta de redução da DI estabelecida até o ano de 2025. Serão necessários mais investimentos nas áreas da educação e saúde, voltados principalmente para o público materno infantil e nas regiões Norte e Nordeste.

## INTRODUÇÃO

A desnutrição infantil (DI) é caracterizada pela deficiência de nutrientes básicos que culmina em déficits de peso e estatura^1^, sendo considerado um problema de saúde pública nos países de média e baixa renda^2^. Índices como peso para idade (P/I) e estatura para idade (E/I) foram adotados pela Organização das Nações Unidas (ONU) para o diagnóstico da DI, sendo o P/I uma medida do estado nutricional recente e a E/I um histórico de nutrição e condições de saúde desde o nascimento, tornando-os requisitos importantes para avaliação da conjuntura socioeconômica e política do país^3-5^.

Em 2000 foram estabelecidos os Objetivos do Desenvolvimento do Milênio (ODM) pelos Estados-membros da ONU, e uma das metas assumidas foi a redução da prevalência mundial de atraso no crescimento de crianças <5 anos de idade à metade, até o ano de 2015^3^. Desde então, a segurança alimentar e nutricional tornou-se alvo das políticas públicas de combate à miséria e DI no Brasil^6^, e a prevalencia de DI reduziu de 13,5% em 1996 para 6,8% em 2007 — atribuída à expansão da cobertura de saúde, educação, saneamento e aumento da renda das famílias^7^.

A partir do sucesso no alcance de muitas metas dos ODM, em 2012, foi instituída a Agenda 2030, com 17 Objetivos do Desenvolvimento Sustentável (ODS), vigorando a partir de 2016^8,9^. Uma das metas internacionais é, até 2030, acabar com todas as formas de desnutrição, e até 2025 atingir as metas internacionais sobre atraso no crescimento (reduzir 40%) e baixo peso (reduzir e manter <5%) em crianças <5 anos^9^. As metas nacionais foram ajustadas pelos especialistas brasileiros para E/I <3%^10,11^. Ressalta-se que o baixo P/I é uma condição aguda que pode mudar frequentemente e rapidamente, o que torna difícil gerar tendências confiáveis ao longo do tempo^12^ enquanto o atraso de crescimento refere-se a uma condição crônica, independentemente da etnia, *status* socioeconômico e tipo de alimentação da criança^13^.

A crise socioeconômica e política que se instalou no país em 2014 impactou no aumento da pobreza, na adoção de medidas de austeridades fiscais, no aumento do preço dos alimentos e na redução das medidas de proteção social aos grupos mais vulneráveis socioeconomicamente, contribuindo para o aumento da DI^14^. Em 2019, a prevalência de déficit estatural chegou a 13,4%, reduzindo-se para 11,7% em 2022, sendo as menores prevalências observadas no Sul (9%) e Sudeste (11,5%), e as maiores no Norte (15,4%) e Nordeste (12,9%), segundo dados do Sistema de Vigilância Alimentar e Nutricional (SISVAN)^15^. Esse sistema apresenta dados da população atendida na Atenção Primária à Saúde (APS) e de beneficiários de programas de transferência de renda^5^, visto que o acompanhamento nutricional é parte de suas condicionalidades^16^.

Existe a necessidade de monitoramento dos indicadores dos ODS para o alcance da meta de redução da DI até 2025, por ser um problema persistente no Brasil. Ademais, a DI apresenta disparidades regionais e consiste em um complexo processo influenciado por causas intrínsecas (fisiológicas) e extrínsecas (alimentação, moradia, meio ambiente, renda familiar, escolaridade dos pais e acesso a bens, serviços essenciais^17^ e assistência materno-infantil^18^) que integram vários indicadores dos ODS^19^. No Brasil, não há estudos que tenham investigado as interações entre os indicadores dos ODS e a prevalência de DI.

A aplicação de métodos estatísticos robustos, como árvore de classificação e regressão (ACR)^20^, possibilitam identificar a interação de distintos fatores sobre a prevalência de DI. A partir desta análise, é possível identificar os indicadores dos ODS determinantes da DI em crianças <5 anos nos municípios brasileiros, bem como seu efeito individual e cumulativo nesta prevalência.

Este estudo teve como objetivo selecionar os indicadores dos ODS determinantes da DI no Brasil mediante o uso da ACR no ano de 2022 e monitorar o alcance das metas da DI, por região. Os achados deste estudo podem fornecer informações que direcionem tomadas de decisão na implementação de políticas públicas sociais e de saúde.

## MÉTODOS

Estudo ecológico que utilizou dados do Índice de Desenvolvimento Sustentável das Cidades — Brasil (IDSC-BR) para acompanhamento da implementação dos ODS. O IDSC-BR utiliza dados produzidos por fontes nacionais como o Instituto Brasileiro de Geografia e Estatística (IBGE), Departamento de Informática do Sistema Único de Saúde (Datasus), Instituto Nacional de Estudos e Pesquisas Educacionais Anísio Teixeira (Inep), Instituto de Pesquisa e Economia Aplicada (Ipea); órgãos produtores de informação; e executores de políticas governamentais ligados à ONU^11^. Os dados utilizados neste estudo foram publicados em 2022 e estão disponíveis no site do IDSC-BR (https://www.cidadessustentaveis.org.br/).

Neste estudo, os 5.570 municípios brasileiros^21^ foram avaliados pelos 100 indicadores dos 17 ODS, dentre eles a DI, cujos dados são proveniente do SISVAN^5^. A variável dependente foi a prevalência de DI em crianças <5 anos, mensurada pelo índice E/I, enquanto as variáveis independentes foram os outros 99 indicadores dos ODS (Quadro 1).

**Quadro 1:** Indicadores dos Objetivos do Desenvolvimento Sustentável e respectivos pontos de corte.

Objetivo do Desenvolvimento Sustentável	Indicador	Valor alvo (%)	Limiar verde (%)	Limiar vermelho (%)	Limite inferior (%)
1	Famílias inscritas no Cadastro Único para programas sociais (%)	96	87	64	48
1	Percentual de pessoas inscritas no Cadastro Único que recebem Bolsa Família	96,6	80,5	42,82	22,96
1	Percentual de pessoas abaixo da linha da pobreza no Cadastro Único pós Bolsa Família	0	21,48	41,59	92,8
1	Pessoas com renda de até 1/4 do salário mínimo (%)	0,18	5,74	4,45	15,45
2	Obesidade infantil (%)	0	5	10	20
2	Baixo peso ao nascer (%)	0	6	11	13
2	Desnutrição infantil (%)	0	1	3	5
2	Produtores de agricultura familiar com apoio do PRONAF (%)	100	75	55	6
3	Cobertura de vacinas (%)	100	95	60	40
3	Mortalidade infantil (crianças menores de 1 ano) (mil nascidas vivas)	0	12	19	45
3	Mortalidade materna (mil nascidos vivos)	0	0,61	3,21	6,7
3	Mortalidade na infância (crianças menores de 5 anos de idade) (mil nascidas vivas)	0	25	37	50
3	Mortalidade neonatal (crianças de 0 a 27 dias) (mil nascidas vivas)	0	12	20	36
3	Orçamento municipal para a saúde (em reais, per capita)	4.680	1300	476	395
3	População atendida por equipes de saúde da família (%)	100	86	60	0
3	Pré-natal insuficiente (%)	0	10	38	59
3	Unidades Básicas de Saúde (mil habitantes)	1,89	0,55	0,15	0
3	Esperança de vida ao nascer (anos)	79	75	71	67
3	Gravidez na adolescência (%)	0	9,98	23,46	30,81
4	Índice de Desenvolvimento da Educação Básica (IDEB) - anos iniciais (IN)	8,98	6,65	4,67	3,8
4	Jovens com ensino médio concluído até os 19 anos de idade (%)	100	70	42	5
4	Analfabetismo na população com 15 anos ou mais (%)	0	3	17	30
4	Crianças e jovens de 4 a 17 anos na escola (%)	100	95	87	82
5	Mulheres jovens de 15 a 24 anos de idade que não estudam nem trabalham (%)	0,83	20,46	39,4	47,06
5	Desigualdade de salário por sexo (salário de mulheres/salário de homens)	1	0,9	0,6	0,5
6	Doenças relacionadas ao saneamento ambiental inadequado (100 mil habitantes)	0	136,21	367,4288361	967,12
6	População atendida com serviço de água (%)	100	85	53	0
6	População atendida com esgotamento sanitário (%)	100	70	50	0
6	População atendida com coleta domiciliar de resíduos sólidos urbanos (%)	100	80	60	0
7	Domicílios com acesso à energia elétrica (%)	100	99	90	80
8	População ocupada entre 10 e 17 anos (%)	0	7,59	25,93	41,32
8	PIB per capita (R$ per capita)	56.000	38.000	23.000	7.300
8	Desemprego (taxa)	0	3	10,27	15,57
9	Investimento público em infraestrutura como proporção do PIB (%)	15	10	5	0,6
9	Participação dos empregos em atividades intensivas em conhecimento e tecnologia (%)	43,28	14,3	1,92	0
10	Renda municipal apropriada pelos 20% mais pobres (%)	20	10	7	1,5
10	Coeficiente de Gini (IN)	0,275	0,3	0,4	0,63
10	Razão mortalidade infantil (negros/não negros)	1	1	1,44	2,08
10	Acesso a equipamentos da atenção básica à saúde	0	2	30	100
11	População residente em aglomerados subnormais (%)	0	0,8	5	22
11	Domicílios em favelas (%)	0	1,04	5,55	13,12
12	Resíduos domiciliares per capita (Ton/Hab/Ano)	1	1,5	2	3,2
17	Investimento público (R$ per capita)	2.253,88	563,255	239,105	60,79
17	Total de receitas arrecadadas (%)	51,35	19,73	3,9	1,19

PRONAF: Programa Nacional de Fortalecimento da Agricultura Familiar; PIB: produto interno bruto.

Segundo o IDSC-BR, a nomenclatura e categorização preconizada para os indicadores dos ODS são: valor alvo (representado pela cor verde), limiar verde (cor amarela), limiar vermelho (cor laranja) e limite inferior (cor vermelha). Ainda considera metas alcançadas a junção do valor alvo e limiar verde, que foram, didaticamente, representadas neste estudo apenas pela cor verde; e metas não alcançadas a junção do limiar vermelho e limite inferior, representados pela cor vermelha^11^. Os valores estabelecidos pelos ODS para avaliar a prevalência de DI foram: <1% (valor alvo); 1% a <3% (limiar verde); 3% a <5% (limiar vermelho) e ≥5% (limite inferior)^11^ (https://www.cidadessustentaveis.org.br/methodology).

Na análise descritiva, as variáveis categóricas foram apresentadas em frequências absolutas e relativas enquanto as contínuas, por médias e erros-padrão da média amostral (EP).

Para a seleção dos indicadores determinantes da prevalência de DI, utilizou-se a ACR^20^. Construída a árvore, calculou-se a sensibilidade para predizer as principais variáveis independentes determinantes da DI. A ACR é um método que divide os dados em segmentos os mais homogêneos possíveis em relação a variável desfecho (prevalência de DI). Um nó é considerado homogêneo quando todos os casos possuem mesmo valor para o desfecho mediante um determinante específico^20^. Foi realizado teste qui-quadrado para verificar a significância estatística dessas correlações e adotado nível de significância de 95%. As análises descritivas e a árvore de classificação foram realizadas utilizando o *software* R, versão 4.3.0.

Este estudo dispensa Termo de Consentimento Livre e Esclarecido e submissão ao Comitê de Ética em Pesquisa por utilizar dados do IDSC-BR, que não contemplam informações sigilosas e são de livre acesso. Foram respeitadas as questões éticas, diretrizes e normas que regulamentam a pesquisa no Brasil.

## RESULTADOS

A distribuição da DI no Brasil, conforme o painel de cores dos ODS, e o alcance de suas metas são apresentados na Figura 1. É nítida a concentração de municípios que atingiram a meta da prevalência de DI nas regiões Sul e Sudeste: 76,5% dos municípios, sendo que 16,2% foram classificados como limiar vermelho e 7,3% como limite inferior (Tabela 1).

**Figura 1:** Distribuição da desnutrição infantil e do alcance das metas estabelecidas pelos Objetivos do Desenvolvimento Sustentável nos municípios brasileiros, segundo a classificação dos Índices do Desenvolvimento Sustentável do Brasil, 2022.

**Tabela 1:** Caracterização da desnutrição infantil e seus determinantes, no Brasil e regiões, de acordo com os Índices de Desenvolvimento Sustentável do Brasil, 2022.

Indicadores		Limites ODS	Brasil n=5.570*	Centro-Oeste n=467*	Nordeste n=1.794*	Norte n=450*	Sudeste n=1.668*	Sul n=1.191*
Desnutrição infantil (%)								
	Valor alvo	≥0 e <1	1.776 (31,9)	129 (27,6)	309 (17,2)	61 (13,6)	600 (36)	677 (56,9)
	Limiar verde	≥1 e <3	2.484 (44,6)	179 (38,4)	947 (52,8)	241 (53,6)	764 (45,8)	353 (29,6)
	Limiar vermelho	≥3 e <5	903 (16,2)	94 (20,1)	393 (21,9)	111 (24,6)	211 (12,6)	94 (7,9)
	Limite inferior	≥5	407 (7,3)	65 (13,9)	145 (8,1)	37 (8,2)	93 (5,6)	67 (5,6)
Analfabetismo ≥15 anos idade (%)								
	Valor alvo	≥0 e <3	102 (1,8)	0 (0)	0 (0)	0 (0)	16 (1)	86 (7,2)
	Limiar verde	≥3 e <17	3.256 (58,5)	411 (88,2)	125 (7)	237 (52,8)	1.406 (84,3)	1.077 (91)
	Limiar vermelho	≥17 e <30	1.532 (27,5)	55 (11,8)	1.032 (57,5)	188 (41,9)	232 (13,9)	25 (2,1)
	Limite inferior	≥30	675 (12,2)	0 (0)	637 (35,5)	24 (5,3)	14 (0,8)	0 (0)
Pré-natal insuficiente (%)								
	Valor alvo	≥0 e <10	614 (11)	38 (8,1)	85 (4,7)	4 (0,9)	216 (12,9)	271 (22,7)
	Limiar verde	≥10 e <38	4.164 (74,8)	362 (77,6)	1.337 (74,6)	195 (43,3)	1.384(83)	886 (74,4)
	Limiar vermelho	≥38 e <59	653 (11,7)	65 (13,9)	312 (17,4)	178 (39,6)	66 (4)	32 (2,7)
	Limite inferior	≥59	139 (2,5)	2 (0,4)	60 (3,3)	73 (16,2)	2 (0,1)	2 (0,2)
Baixo peso ao nascer (%)								
	Valor alvo	≥0 e <6	1.243 (22,3)	144 (30,8)	438 (24,4)	129 (28,7)	275 (16,5)	257 (21,6)
	Limiar verde	≥6 e <11	3.476 (62,4)	268 (57,4)	1.191 66,4)	290 (64,4)	1.057(63,4)	670 (56,2)
	Limiar vermelho	≥11 e <13	439 (7,9)	29 (6,2)	102 (5,7)	14 (3,1)	187 (11,2)	107 (9)
	Limite inferior	≥13	412 (7,4)	26 (5,6)	63 (3,5)	17 (3,8)	149 (8,9)	157 (13,2)
Mulheres jovens entre 15-24 anos que não estudam nem trabalham (%)								
	Valor alvo	≥0,83 e 20,46	850 (15,4)	19 (4,1)	18 (1)	4 (0,9)	189 (11,3)	620 (52,2)
	Limiar verde	≥20,46 e 39,4	3.876 (69,6)	377 (80,9)	1.262(70,3)	323 (71,9)	1.374(82,4)	540 (45,5)
	Limiar vermelho	≥39,4 e 47,06	699 (12,5)	59 (12,6)	423 (23,6)	97 (21,6)	97 (5,8)	23 (1,9)
	Limite inferior	≥47,06	140 (2,5)	11 (2,4)	91 (5,1)	25 (5,6)	8 (0,5)	5 (0,4)
População ocupada entre 10-17 anos								
	Valor alvo	≥0 e <7,59	547 (9,8)	19 (4,1)	294 (16,4)	37 (8,2)	178 (10,7)	19 (1,6)
	Limiar verde	≥7,59 e 25,93	4.317 (77,5)	413 (88,4)	1.397(77,9)	376 (83,6)	1.424(85,4)	707 (59,4)
	Limiar vermelho	≥25,93 e 41,32	566 (10,2)	34 (7,3)	97 (5,4)	34 (7,5)	62 (3,7)	339 (28,4)
	Limite inferior	≥41,32	140 (2,5)	1 (0,2)	6 (0,3)	3 (0,67)	4 (0,2)	126 (10,6)

* número de municípios. ODS: Objetivos do Desenvolvimento Sustentável; Valor alvo: valor que reflete o melhor desempenho do município, estabelecido como meta dos ODS para o indicador referido; Limiar verde: valor do indicador, a partir do qual considera-se que o município atingiu a meta dos ODS; Limiar vermelho: valor que denota distância do cumprimento da meta desejada pelo município para o indicador do ODS; Limiar inferior: valor que reflete o pior desempenho do município para o indicador em estudo.

A região Sul apresentou 86,5% de municípios que atingiram a meta de redução da DI, seguida pela região Sudeste com 81,8%. Nas regiões Nordeste, Norte e Centro-Oeste, os percentuais de municípios que atingiram a meta foram 70, 67,1 e 66%, respectivamente (Tabela 1).

Os seguintes indicadores foram selecionados pela ACR como determinantes da DI no Brasil: analfabetismo na população ≥15 anos (Analfabetismo ≥15 anos), pré-natal insuficiente (PNI), baixo peso ao nascer (BPN), mulheres jovens de 15 a 24 anos que não estudam nem trabalham (Mulheres 15-24 anos Nem-Nem) e população ocupada de 10 a 17 anos de idade (Pop-Ocupada 10-17 anos). Estimou-se o efeito cumulativo do aumento desses indicadores sobre a prevalência de DI (Figura 2).

**Figura 2:** Árvore de classificação e regressão e a correlação entre os indicadores dos Objetivos do Desenvolvimento Sustentável determinantes da desnutrição infantil. Brasil, 2022.

A prevalência de municípios que atingiram a meta de redução do analfabetismo ≥15 anos foi de 60,3% e da meta de superação do PNI, 85,8%. As regiões Sul, Centro-Oeste e Sudeste apresentaram maior número de municípios que atingiram a meta. Entretanto, 93% dos municípios do Nordeste não atingiram a meta de redução do analfabetismo, e 55,8% dos municípios do Norte não atingiram a meta de superação do PNI (Tabela 1).

No Brasil, 84,7% dos municípios atingiram a meta de superação do BPN. As regiões Sul e Sudeste apresentaram maior número de municípios que não atingiram a meta: 22,3 e 20,1%, respectivamente (Tabela 1).

Dentre os municípios brasileiros, 85% atingiram a meta de redução do indicador Mulheres 15-24 anos Nem-Nem. As regiões Nordeste e Norte apresentaram maior número de municípios que não atingiram a meta para esse indicador (28,7 e 27,2%, respectivamente). Atingiram a meta de redução da Pop-Ocupada 10-17 anos 87,3% dos municípios, e a região Sul apresentou maior percentual de municípios que não alcançaram a meta (39%) (Tabela 1).

A análise da ACR mostrou que em 2.713 municípios, nos quais o percentual de Analfabetismo ≥15 anos foi <13%, a prevalência de DI foi de 1,73% (EP±0,04). Em 2.017 municípios, onde o percentual de Analfabetismo ≥15 anos foi ≥13%, associado ao PNI <34%, a prevalência de DI elevou-se para 2,37% (EP±0,05). Em 596 municípios, nos quais os percentuais de Analfabetismo ≥ 15 anos foram >13%, PNI ≥34% e BPN <9%, a prevalência de DI foi de 2,86% (EP±0,09). Em 122 municípios que, além do Analfabetismo ≥15 anos >13%, do PNI ≥34%, BPN ≥9%, Mulheres 15-24 anos Nem-Nem contabilizaram <35%, a prevalência de DI aumentou para 2,89% (EP±0,22) (Figura 2).

Em 115 municípios que apresentaram >13% de Analfabetismo ≥15 anos, PNI ≥34%, BPN ≥9%, Mulheres 15-24 anos Nem-Nem ≥35% e Pop-Ocupada 10-17 anos <19%, a prevalência de DI foi 4,07% (EP±0,40). Em sete municípios, quando a Pop-Ocupada 10-17 anos foi ≥19% e todas as demais iniquidades se mantiveram, a prevalência de DI aumentou para 15,4% (EP±5,54) (Figura 2).

## DISCUSSÃO

Esse estudo evidenciou que a meta dos ODS para redução da DI foi atingida por 76,5% dos municípios brasileiros, com destaque para as regiões Sul e Sudeste. O Centro-Oeste apresentou maior percentual de municípios que não alcançaram essa meta. Cinco indicadores dos ODS foram selecionados como determinantes da DI segundo percentuais e ordem de impacto (de acordo com o surgimento do indicador na ACR): Analfabetismo ≥15 anos, PNI, BPN, Mulheres 15-24 anos Nem-Nem e Pop-Ocupada 10-17 anos.

O fato de a região Centro-Oeste apresentar prevalência superior ao Norte, emerge questionamentos sobre a subnotificação e incompletude de dados nessa região, recorrentes em pesquisas nacionais, podendo mascarar resultados e impactar em tomadas de decisões^22^.

A prevalência de DI por E/I no Brasil, segundo dados da PNDS/2007 (Pesquisa Nacional de Demografia e Saúde), foi de 6,8%^7^, atingindo a meta estabelecida pelos ODM^3^. No entanto, dados do SISVAN revelaram valores maiores, variando de 15,1% (2008) a 13,4% (2019), com pequena redução em 2022 (11,7%), sendo maior no Norte (15,4%) e Nordeste (12,9%)^15^. Por outro lado, o Estudo Nacional de Alimentação e Nutrição Infantil (Enani/2019), representativo da população brasileira, revelou prevalência de 7% de baixa E/I, sendo maior no Norte (8,4%), Sudeste (7,3%) e Sul (7%)^23^.

Devido à lacuna entre a PNDS/2007 e o ENANI/2019, desde o ano de 2008, os dados do SISVAN foram utilizados para monitoramento dessas metas^5^. O SISVAN apresenta dados de todas as regiões e possibilita o estudo da população atendida na APS^24^ e dos beneficiários de Programa Bolsa Família (PBF)^5,16^. Posteriormente, os dados do Enani/2019 passaram a ser incorporados aos relatórios de acompanhamento dos ODS para análise desses indicadores^25^.

Independentemente das diferenças regionais, a crise socioenômica e política que se instalou no Brasil desde 2014 impactou no aumento da pobreza; aumento do preço dos alimentos; na adoção de medidas de austeridades fiscais e redução das medidas de proteção social, contribuindo para o aumento da DI^14^.

Apesar da pandemia por COVID-19, em 2020, as medidas de proteção adotadas pelo poder público, em todas as suas esferas — por meio da criação do Auxilio Emergencial; Programa Emergencial de Manutenção do Emprego e Renda^26^; e dos incentivos financeiros específicos para a APS no enfrentamento da má nutrição, com foco em crianças e gestantes —, contribuiram para mitigar o efeito da insegurança alimentar e atenuar o impacto da pandemia na DI^27^.

A gravidade do analfabetismo funcional foi evidenciada no Nordeste e Norte, onde grande parcela dos municípios não atingiu a meta, se opondo às demais regiões. Um estudo no sul da África identificou que a prevalência de DI variou entre 3,4 e 30,2%, tendo sido associada ao analfabetismo^28^. Este último pode desencadear menos oportunidades de trabalho, menor renda e dificuldade na aquisição de alimentos e no acesso aos serviços de saúde, contribuindo para a DI^7^.

A meta de redução do analfabetismo não foi concretizada pelo Plano Nacional de Educação 2014-2024 devido a não implementação de políticas públicas de educação voltadas para o público ≥15 anos^29^. Além disso, não foram estabelecidos mecanismos para compatibilizar a jornada de trabalho, nem programa de transferência de renda específico^30^. Apenas em 2024 foi instituído o Programa Pé de Meia, voltado para estudantes de 14 a 24 anos de escolas públicas e aos pertencentes à categoria de Educação de Jovens e Adultos (EJA), de 19 a 24 anos^31^.

A meta de superação do PNI foi atingida por muitos municípios das regiões brasileiras, com exceção da região Norte. A cobertura do pré-natal no Brasil desde 2013^32^ até 2019^33^ foi de 89%. Ressalta-se que o Norte apresenta maior nível de precariedade de serviços, baixa cobertura de pré-natal e piores indicadores de qualidade^34^, relacionados à abrangência territorial, dificuldade de acesso aos serviços de saúde e baixa fixação de profissionais na região^35^.

O acompanhamento nutricional durante o pré-natal é essencial para a prevenção do ganho de peso inadequado e controle de intercorrências^36^ que podem predispor a gestante ao parto prematuro, e a criança à restrição de crescimento intrauterino (RCIU) e ao BPN^37^. A gestante deve ser orientada quanto à amamentação e alimentação adequada para prevenir a DI^38^.

Nesta pesquisa, elevada frequência de municípios brasileiros do Norte, Nordeste e Centro-Oeste superaram a meta de BPN; o mesmo não ocorreu no Sul e Sudeste. As prevalências de BPN são 37,7% no Sul e 13,5% no Sudeste^39^.

O paradoxo do BPN foi observado nas regiões Norte e Sul. Este pode ser atribuído à melhora da assistência pré-natal nas regiões mais desenvolvidas com concomitante redução da prevalência de natimortos e aumento do BPN^40^, como observado na região Sul. O oposto foi observado no Norte, com menor prevalência de BPN e alto percentual de municípios com PNI. As possíveis explicações seriam as altas taxas de subnotificação de nascidos vivos no Norte^41^ e a elevada taxa de partos cesáreos no Sul^42^, relacionados à interrupção de gestações de risco, com consequente redução da idade gestacional, aumento da prematuridade e viabilidade de recém-nascidos com extremo baixo peso^43^.

O Brasil apresenta uma das maiores proporções de cesariana no mundo. Em 2019, 56,3% dos partos foram cesarianas e realizados principalmente nas regiões Centro-Oeste, Sudeste e Sul^44^, ressaltando as desigualdade no país. Posteriormente, estudos apontaram tendência de aumento no Norte e Nordeste, e declínio no Sul e Sudeste^42^. Apesar do declínio, a região Sul ainda apresenta maiores prevalências, e o Brasil permanece com taxas de cesariana muito acima do recomendado^43^.

Políticas públicas desenvolvidas desde a década de 1980, que culminaram com a implantação do Programa Rede Cegonha^45^, foram essenciais para a melhora de práticas importantes relacionadas ao parto e puerpério no Brasil. Outras políticas e estratégias relacionadas à proteção do aleitamento materno e introdução à alimentação complementar adequada e saudável foram essenciais para proteção da saúde infantil, especiamente dos lactentes, pois observa-se que as intercorrências no período neonatal estão mais relacionadas à gestação e ao parto e, após este período, estão mais relacionadas ao contexto socioeconômico da criança^46^.

A meta de redução do indicador Mulheres 15-24a Nem-Nem foi atingida por 85% dos municípios, com piores resultados no Norte e Nordeste. Em 2018, 23% dos jovens encontravam-se nesta situação, sendo a maioria mulheres de baixa renda^10^. Os motivos estão relacionados a habilidades cognitivas, obrigações domésticas e falta de políticas públicas que possam mitigar a desigualdade de gênero e elevar as perspectivas de superação da pobreza^47^. A baixa escolaridade, baixa renda e gestação na adolescência estão mais relacionadas à RCIU e a maior risco de gerar crianças desnutridas^48,49^. O investimento no capital humano^2^ dessas mulheres pode contribuir para o melhor planejamento familiar e a redução da DI^50^.

A escolaridade das mulheres é um forte preditor de saúde e sobrevivência de seus filhos, pois influencia no acesso aos serviços de saúde, cuidados em situações de adoecimento, às melhores oportunidades de trabalho e à geração de renda para os cuidados com a criança^51^. As mães tendem a gerenciar melhor os gastos familiares, direcionando-os para alimentação, vestuários e material escolar^52^.

A região Sul é a mais afetada pelo trabalho infantil, e com elevada prevalência do indicador Pop-Ocupada 10-17 anos. O trabalho infantil pode ocasionar danos na saúde física e mental de crianças e adolescentes, além do risco de adesão a hábitos de vida não saudáveis e desenvolvimento de doenças crônicas não transmissíveis^53^. Também perpetua a baixa escolaridade, renda^54^ e DI^46^. Paradoxalmente, na região Sul, esse cenário pode estar relacionado à cultura que contempla o trabalho como ferramenta de educação^55^, pois não impactou negativamente nas taxas de analfabetismo, pré-natal, desigualdades de gênero e DI.

Nas demais regiões, para incremento da renda familiar, o trabalho infantil contribui para altas taxas de retenção, desistência e evasão escolar, necessitando de implementação de políticas que o desestimule. Lares providos apenas por mulheres aumentam o risco do trabalho infantil quando comparado à provisão paterna ou de ambos. Quanto maior a escolaridade e renda dos pais, menor o risco de abandono escolar^55,56^.

Os pontos de corte para cada indicador de DI, identificados pela ACR, estavam dentro da meta proposta pelos ODS. Apesar disso, esses contribuíram para o aumento da prevalência de DI. Ademais, o efeito cumulativo (efeito combinado desses indicadores na ordem que surgem na ACR) foi ainda mais impactante no aumento da prevalência de DI.

Alguns estudos também observaram o efeito cumulativo de indicadores de iniquidades sociais e de saúde associados à DI. O maior nível de escolaridade materna está associado à redução da DI^57,58^ e a maior adesão ao pré-natal^59^. No Rio Grande do Sul, foi encontrada associação entre pré-natal adequado e redução do BPN^60^. Em Pernambuco, observou-se associação da DI a menor escolaridade materna e BPN^61^. Uma revisão realizada na região Norte associou a DI à baixa escolaridade materna e a possuir pai/padrasto não alfabetizado^62^.

A complexidade do desfecho deste estudo é contemplada por meio do impacto que cada indicador, identificado como determinante da DI, influencia na sua prevalência, bem como pelas interrelações e interdependências que esses indicadores exercem na sucessão de eventos que favoreceram o aumento da prevalência de DI no Brasil.

As desigualdades regionais observadas foram estreitamente relacionadas ao alcance ou distanciamento das metas dos indicadores estudados, o que as tornaram vulneráveis ao aumento da DI.

Este estudo apresenta como limitações a utilização do banco de dados do IDSC-BR, que: considerou informações de anos próximos a 2022 para os indicadores, visto que alguns não traziam informações atualizadas; e utiliza dados do SISVAN para o monitoramento da DI. No entanto, as informações obtidas por meio do IDSC-BR constituem-se uma base de dados robusta da população brasileira, construída a partir da junção de informações de diversos órgãos e agências ligados à ONU^11^. Além disso, embora o SISVAN apresente falhas quanto à coleta dos dados, suas informações são consideradas oficiais para fins estatísticos devido a sua cobertura e ao número de informações por estado^14^.

Como aspectos positivos, ressalta-se que todos os indicadores de monitoramento dos ODS, em todos os municípios brasileiros — dispostos no banco de dados —, foram utilizados neste estudo, possibilitando uma investigação ampla do problema em questão. Além disso, foi utilizada a ACR para selecionar os indicadores determinantes da DI e estabelecer os pontos de corte de forma isolada e cumulativa.

Conclui-se que os indicadores de monitoramento dos ODS determinantes da DI no Brasil, isoladamente e em conjunto, foram Analfabetismo ≥15 anos, PNI, BPN, Mulheres 15-24 anos Nem-Nem e Pop-Ocupada 10-17 anos. O impacto desses indicadores sobre a prevalência de DI apresentou média de 1,73%, quando a prevalência do Analfabetismo ≥15 anos foi menor que 13%; até a média de 15,1%, quando todos os demais indicadores ultrapassaram os percentuais identificados pela ACR. Além disso, a maioria dos municípios brasileiros atingiu as metas para os indicadores selecionados pela ACR.

Todos os indicadores determinantes da DI estão relacionados à pobreza e às desigualdades sociais, dificultando o alcance da meta para a DI no país. Torna-se necessária a implantação e implementação de políticas socioeconômicas multidimensionais, pelos gestores de todas as esferas, que melhorem, em curto e longo prazos, a renda das famílias vulneráveis e o acesso à educação e aos serviços de saúde, especialmente para adolescentes/jovens e mulheres, de forma que minimizem e detenham o aumento das desigualdades existentes.

Além disso, as regiões Norte e Nordeste do país também devem ser priorizadas quanto aos investimentos para a atenção à saúde materno infantil, na qual haja acompanhamento da mulher desde o pré-natal, passando pelo parto, puerpério e os primeiros anos da vida da criança — consequentemente minimizando os riscos à saúde.
